# Supplementary material for: Seroprevalence of SARS-CoV-2 antibodies in Seattle, Washington: October 2019–April 2020
Source: PLoS One. 2021 May 27;16(5):e0252235. doi: 10.1371/journal.pone.0252235 (PMC8158900; doi:10.1371/journal.pone.0252235)
Supplement: Attachment — Submitted filename: Response to Reviewers.docx [file pone.0252235.s002.docx]

**Response to Reviewers**

PONE-D-21-07261
Seroprevalence of SARS-CoV-2 Antibodies in Seattle, Washington:

October 2019–April 2020
PLOS ONE

Dear Dr. Denise J McCulloch

Thank you for submitting your manuscript to PLOS ONE. After careful consideration, we feel that it has merit but does not fully meet PLOS ONE’s publication criteria as it currently stands. Therefore, we invite you to submit a revised version of the manuscript that addresses the points raised during the review process. Both reviewers ask you to enrich your discussion and to compare your data to other previous records and publications. Please submit your revised manuscript by 7th of May, 2021. If you will need more time than this to complete your revisions, please reply to this message or contact the journal office at plosone@plos.org. When you're ready to submit your revision, log on to https://www.editorialmanager.com/pone/ and select the 'Submissions Needing Revision' folder to locate your manuscript file.

Thank you for reviewing our manuscript. We have enriched the discussion and compared our data to other publications.

Please include the following items when submitting your revised manuscript:

- A rebuttal letter that responds to each point raised by the academic editor and reviewer(s). You should upload this letter as a separate file labeled 'Response to Reviewers'.
- A marked-up copy of your manuscript that highlights changes made to the original version. You should upload this as a separate file labeled 'Revised Manuscript with Track Changes'.
- An unmarked version of your revised paper without tracked changes. You should upload this as a separate file labeled 'Manuscript'.

We have labeled the files as requested and uploaded them to the editorial manager website.

If you would like to make changes to your financial disclosure, please include your updated statement in your cover letter. Guidelines for resubmitting your figure files are available below the reviewer comments at the end of this letter.

We have updated our financial disclosure and have included the updated statement in our cover letter.

If applicable, we recommend that you deposit your laboratory protocols in protocols.io to enhance the reproducibility of your results. Protocols.io assigns your protocol its own identifier (DOI) so that it can be cited independently in the future. For instructions see: <http://journals.plos.org/plosone/s/submission-guidelines#loc-laboratory-protocols>. Additionally, PLOS ONE offers an option for publishing peer-reviewed Lab Protocol articles, which describe protocols hosted on protocols.io. Read more information on sharing protocols at <https://plos.org/protocols?utm_medium=editorial-email&utm_source=authorletters&utm_campaign=protocols>.

We look forward to receiving your revised manuscript.

Kind regards,

Gheyath K. Nasrallah, PhD
Academic Editor
PLOS ONE

Journal Requirements:

Please review your reference list to ensure that it is complete and correct. If you have cited papers that have been retracted, please include the rationale for doing so in the manuscript text, or remove these references and replace them with relevant current references. Any changes to the reference list should be mentioned in the rebuttal letter that accompanies your revised manuscript. If you need to cite a retracted article, indicate the article’s retracted status in the References list and also include a citation and full reference for the retraction notice.

We have reviewed the reference list and ensured it is complete and correct.

When submitting your revision, we need you to address these additional requirements.

1. Please ensure that your manuscript meets PLOS ONE's style requirements, including those for file naming. The PLOS ONE style templates can be found at

https://journals.plos.org/plosone/s/file?id=wjVg/PLOSOne_formatting_sample_main_body.pdf and

[https://journals.plos.org/plosone/s/file?id=ba62/PLOSOne_formatting_sample_title_authors_affiliations.pdf](about:blank)

We have updated the formatting to meet PLOS ONE style requirements.

2. Thank you for stating the following in the Competing Interests section:

" I have read the journal's policy and the authors of this manuscript have the following competing interests:
Dr. Chu reported consulting with Ellume, Pfizer, The Bill and Melinda Gates Foundation, Glaxo Smith Kline, and Merck. She has received research funding from Sanofi Pasteur and Ellume, and support and reagents from Cepheid outside of the submitted work.

Dr. Jackson reports receiving research funding from Sanofi Pasteur.

Denise J. McCulloch, James P. Hughes, Sandra Lester, Lisa Mills, Brandi Freeman, Mohammad Ata Ut Rasheed, and Natalie J. Thornburg have declared that no competing interests exist."

We note that one or more of the authors are employed by a commercial company: Kaiser Permanente, Synergy America, Inc, Eagle Global Scientific, LLC.

2.1. Please provide an amended Funding Statement declaring this commercial affiliation, as well as a statement regarding the Role of Funders in your study. If the funding organization did not play a role in the study design, data collection and analysis, decision to publish, or preparation of the manuscript and only provided financial support in the form of authors' salaries and/or research materials, please review your statements relating to the author contributions, and ensure you have specifically and accurately indicated the role(s) that these authors had in your study. You can update author roles in the Author Contributions section of the online submission form.

We have provided in our cover letter an updated funding statement, which reflects those authors’ employment by commercial companies.

Please also include the following statement within your amended Funding Statement.

“The funder provided support in the form of salaries for authors [insert relevant initials], but did not have any additional role in the study design, data collection and analysis, decision to publish, or preparation of the manuscript. The specific roles of these authors are articulated in the ‘author contributions’ section.”

If your commercial affiliation did play a role in your study, please state and explain this role within your updated Funding Statement.

We have included this statement about the role of funders in our updated funding statement.

2.2. Please also provide an updated Competing Interests Statement declaring this commercial affiliation along with any other relevant declarations relating to employment, consultancy, patents, products in development, or marketed products, etc.

Within your Competing Interests Statement, please confirm that this commercial affiliation does not alter your adherence to all PLOS ONE policies on sharing data and materials by including the following statement: "This does not alter our adherence to  PLOS ONE policies on sharing data and materials.” (as detailed online in our guide for authors [http://journals.plos.org/plosone/s/competing-interests](about:blank)) . If this adherence statement is not accurate and  there are restrictions on sharing of data and/or materials, please state these. Please note that we cannot proceed with consideration of your article until this information has been declared.

Please include both an updated Funding Statement and Competing Interests Statement in your cover letter. We will change the online submission form on your behalf.

We have updated our Competing Interests Statement, and have included this statement about sharing of data and materials. This updated Competing Interest Statement is included in our cover letter and in the updated manuscript.

Please know it is PLOS ONE policy for corresponding authors to declare, on behalf of all authors, all potential competing interests for the purposes of transparency. PLOS defines a competing interest as anything that interferes with, or could reasonably be perceived as interfering with, the full and objective presentation, peer review, editorial decision-making, or publication of research or non-research articles submitted to one of the journals. Competing interests can be financial or non-financial, professional, or personal. Competing interests can arise in relationship to an organization or another person. Please follow this link to our website for more details on competing interests: [http://journals.plos.org/plosone/s/competing-interests](about:blank)

3. Please include captions for your Supporting Information files at the end of your manuscript, and update any in-text citations to match accordingly. Please see our Supporting Information guidelines for more information: [http://journals.plos.org/plosone/s/supporting-information](about:blank).

We have included a caption for our supporting information file at the end of the manuscript.

[Note: HTML markup is below. Please do not edit.]

Reviewers' comments:

Reviewer's Responses to Questions

**Comments to the Author**

1. Is the manuscript technically sound, and do the data support the conclusions?

The manuscript must describe a technically sound piece of scientific research with data that supports the conclusions. Experiments must have been conducted rigorously, with appropriate controls, replication, and sample sizes. The conclusions must be drawn appropriately based on the data presented.

Reviewer #1: Yes

Reviewer #2: Yes

2. Has the statistical analysis been performed appropriately and rigorously?

Reviewer #1: Yes

Reviewer #2: N/A

3. Have the authors made all data underlying the findings in their manuscript fully available?

The [PLOS Data policy](http://www.plosone.org/static/policies.action#sharing) requires authors to make all data underlying the findings described in their manuscript fully available without restriction, with rare exception (please refer to the Data Availability Statement in the manuscript PDF file). The data should be provided as part of the manuscript or its supporting information, or deposited to a public repository. For example, in addition to summary statistics, the data points behind means, medians and variance measures should be available. If there are restrictions on publicly sharing data—e.g. participant privacy or use of data from a third party—those must be specified.

Reviewer #1: Yes

Reviewer #2: Yes

4. Is the manuscript presented in an intelligible fashion and written in standard English?

PLOS ONE does not copyedit accepted manuscripts, so the language in submitted articles must be clear, correct, and unambiguous. Any typographical or grammatical errors should be corrected at revision, so please note any specific errors here.

Reviewer #1: Yes

Reviewer #2: Yes

5. Review Comments to the Author

Please use the space provided to explain your answers to the questions above. You may also include additional comments for the author, including concerns about dual publication, research ethics, or publication ethics. (Please upload your review as an attachment if it exceeds 20,000 characters)

**Reviewer #1:** This is an interesting manuscript but it needs to be improved to be of greater interest.

It presents numerous weak points, recognized at the end of the discussion by the authors. To gain consistency it is necessary to compare its results with previous records in other nations and other continents. The strong point of the study is that it documents (with the aforementioned limitations) the null seroprevalence prior to the pandemic outbreak in the United States. It would be interesting to make a comparison with the sequential seroprevalence in the United States with respect to other continents.

**Response:** We have added two paragraphs to the discussion to compare our results with those in other nations and continents. We have included references to data from Europe, the Middle East, and Asia in order to help put our findings into a global context. The added paragraphs are as follows:

The absence of earlier circulation of SARS-CoV-2 in our study is consistent with findings from two studies in Canada examining residual nasopharyngeal samples from December 2019 onward, which did not find evidence of positive SARS-CoV-2 samples prior to late February 2020 [4, 5]. By contrast, however, evidence from Europe suggests that early SARS-CoV-2 may have been circulating in France and Italy since December 2019 [6, 7].

Furthermore, our finding of a SARS-CoV-2 seroprevalence around 1% in early April is consistent with larger serosurveys in the Pacific Northwest of the United States around this time [8, 9], possibly due to rapid and widespread lockdowns imposed early in the pandemic [10]. Similar seropositivity was observed at that time in other locations with early success in controlling the spread of SARS-CoV-2, including Germany (0.97%) [11], Canada (0.7%) [12], Denmark (1.4%) [13] and Korea (0.4%) [14]. This contrasts with significantly higher SARS-CoV-2 antibody positivity in parts of the world that had more cases early on in the pandemic, including other parts of the United States (3.2 – 6.9%) [9], Wuhan, China (4.4%) [15], Spain (4.6%) [16], Geneva (4.8%) [17], Ariano Irpino, Italy (5.6%) [18], and northern Iran (22.2%) [19].

**Reviewer #2**: This is a short report that is effective in providing useful data for the epidemiology of SARS-CoV-2. I would encourage (not necessary, though) the authors to include in the discussion our experience in the town of Ariano Irpino - different strategy, mass screening using serology, stronger approach useful for both screening and epidemiology study. Please refer to https://pubmed.ncbi.nlm.nih.gov/33585828/ in the Discussion.

**Response:** As noted above, we have added additional references to the discussion in order to better compare our findings to the existing literature. We have included both references suggested by Reviewer #2.

6. PLOS authors have the option to publish the peer review history of their article ([what does this mean?](https://journals.plos.org/plosone/s/editorial-and-peer-review-process#loc-peer-review-history)). If published, this will include your full peer review and any attached files.


If you choose “no”, your identity will remain anonymous but your review may still be made public.


**Do you want your identity to be public for this peer review?** For information about this choice, including consent withdrawal, please see our [Privacy Policy](https://www.plos.org/privacy-policy).

Reviewer #1: **Yes:** Jose F Varona

Reviewer #2: **Yes:** Carlo Buonerba

[NOTE: If reviewer comments were submitted as an attachment file, they will be attached to this email and accessible via the submission site. Please log into your account, locate the manuscript record, and check for the action link "View Attachments". If this link does not appear, there are no attachment files.]

While revising your submission, please upload your figure files to the Preflight Analysis and Conversion Engine (PACE) digital diagnostic tool, <https://pacev2.apexcovantage.com/>. PACE helps ensure that figures meet PLOS requirements. To use PACE, you must first register as a user. Registration is free. Then, login and navigate to the UPLOAD tab, where you will find detailed instructions on how to use the tool. If you encounter any issues or have any questions when using PACE, please email PLOS at [figures@plos.org](mailto:figures@plos.org). Please note that Supporting Information files do not need this step.
